# Reliability coefficients for multiple group item response theory models

**DOI:** 10.1111/bmsp.12269

**Published:** 2022-03-01

**Authors:** Björn Andersson, Hao Luo, Kseniia Marcq

**Affiliations:** ^1^ University of Oslo Norway; ^2^ University of Hong Kong Hong Kong SAR China

**Keywords:** confidence intervals, item response theory, multiple groups, reliability

## Abstract

Reliability of scores from psychological or educational assessments provides important information regarding the precision of measurement. The reliability of scores is however population dependent and may vary across groups. In item response theory, this population dependence can be attributed to differential item functioning or to differences in the latent distributions between groups and needs to be accounted for when estimating the reliability of scores for different groups. Here, we introduce group‐specific and overall reliability coefficients for sum scores and maximum likelihood ability estimates defined by a multiple group item response theory model. We derive confidence intervals using asymptotic theory and evaluate the empirical properties of estimators and the confidence intervals in a simulation study. The results show that the estimators are largely unbiased and that the confidence intervals are accurate with moderately large sample sizes. We exemplify the approach with the Montreal Cognitive Assessment (MoCA) in two groups defined by education level and give recommendations for applied work.

## 1 Introduction

The reliability of scores from a psychological scale or test refers to the consistency of the measurement and is an essential component in ensuring the validity of uses of the test scores (American Educational Research Association, American Psychological Association, & National Council on Measurement in Education, 2014). Reliability is generally defined as the ratio of the true score variance to the observed score variance. The true score variance and observed score variance are unknown, however, and need to be estimated from the observed data. In practice, how to estimate the reliability of different scores depends on the data collection method and the modelling framework employed. The present work aims to introduce new approaches to estimate reliability coefficients with multiple‐group item response theory (IRT).

Several reliability coefficients have been proposed in different contexts based on different modelling frameworks. In classical test theory, where sum scores are typically used, the most extensively used measure of reliability is coefficient alpha (Cronbach, 1951). However, coefficient alpha is only equal to the reliability of the sum scores with a tau‐equivalent single‐factor model (Novick & Lewis, 1967). Since its assumptions almost never hold, it is well recognized that coefficient alpha is only a lower bound on the reliability of the sum scores when using a single‐factor model (Sijtsma, 2009). In an IRT, confirmatory factor analysis or structural equation modelling framework, reliability of the sum scores can instead be directly estimated by utilizing the assumptions implicit in these models (Cheng, Yuan, & Liu, 2012; Green & Yang, 2009; Jöreskog, 1971; Kim & Feldt, 2010). For confirmatory factor analysis models, the reliability of the sum scores is equal to what is called composite reliability (Jöreskog, 1971). For IRT models, formulas are available that enable the estimation of the reliability of the sum scores using the estimated model parameters (Kim & Feldt, 2010). While reliability coefficients most often concern the reliability of sum scores, it is also possible to consider the reliability coefficient of other types of scores such as factor scores from factor analysis models or ability estimates from IRT models (Cheng et al., 2012; Kim & Nicewander, 1993; Kim, 2012; Nicewander & Thomasson, 1999). Note here that these reliability coefficients refer to the consistency of measurement of the scores in a population and do not concern the reliability of the sum score or ability estimate of a single person. In IRT it is common to use the maximum likelihood ability estimator since it is an unbiased estimator of the ability when the number of items tends to infinity (Lord, 1980). The reliability coefficient of scores based on this estimator was discussed, for example, in Cheng et al. (2012), Kim (2012), and Lord (1983). Similarly, reliability coefficients for the posterior mean and mode have been considered (Nicewander & Thomasson, 1999). There are also extensions of the various approaches that enable the estimation of reliability with multilevel models (Cho, Shen, & Naveiras, 2019; Geldhof, Preacher, & Zyphur, 2014; Raykov & Penev, 2010).

The reliability of the scores from a scale or test may vary with respect to the population (McDonald, 1999). This means that scores from the same test can be reliable for certain populations while being unreliable for other populations, as discussed by Raykov (2002) in the context of multiple‐group factor analysis. However, although investigations into differences in item characteristics between populations have become increasingly commonplace, differences in the corresponding score reliabilities are not always reflected on. When analysing test data from a heterogeneous population, studies of measurement invariance with respect to test‐taker characteristics such as biological sex, socioeconomic status or age group are often done in order to ensure the appropriateness of using the resulting scores on the test. In these settings, multiple‐group IRT is often used (Balsis, Gleason, Woods, & Oltmanns, 2007; Muthén & Lehman, 1985). When estimating multiple‐group IRT models, assuming that there exists a set of invariant items, it is possible to account for differences in the latent distributions between groups and also differences in item parameters between groups. A consequence of these differences is that the score reliabilities for the groups may be different. The differences in reliability coefficients based on group membership have been considered with multiple‐group IRT in software packages such as *mirt* (Chalmers, 2012), where item parameter estimates from different groups were used when computing the marginal reliability coefficient defined in Thissen and Wainer (2001). However, an exposition of methods for estimating reliability coefficients with IRT multiple‐group models that account for distribution parameters is missing in the literature. Note that scale scores from the same test are often used in multiple ways. Using the scale scores to draw inference of a latent construct for an individual is often done in relation to the average scores in a particular group defined by specific characteristics such as education level or age. In this case groupwise reliability coefficients are of particular interest. However, when using the scale scores as an explanatory variable in a regression model or path analysis model with data from all individuals in a sample, the overall reliability coefficient is the relevant quantity of interest and is one way to properly adjust for measurement error (Cole & Preacher, 2014; Gleser, 1992), besides using, for example, multiple imputation. Thus, in practice, both the groupwise and overall reliability coefficients are important to consider. This study aims to improve the estimation of these reliability coefficients when using multiple‐group IRT models.

Within the framework of IRT, it is possible to estimate the reliability coefficients for different groups in two ways. One approach is to estimate separate IRT models in the different groups and for all groups combined, and estimate the reliability coefficients separately with these models using the existing approaches for single‐group models (Cheng et al., 2012; Kim & Feldt, 2010). Another approach is to use multiple‐group IRT models in order to simultaneously estimate the score reliabilities for each individual group and also for the multiple groups combined. There are three main statistical advantages to estimating the reliability coefficients with a multiple‐group model. First, the efficiency is improved in the estimation of the groupwise reliability coefficients since data from all groups are used to estimate the item parameters. If instead estimating the item parameters, and thus the reliability coefficients, separately in each group, the information from the other groups in the data is disregarded and estimation precision is lost. Second, the estimation accuracy of the overall reliability coefficient (across all groups) is improved compared to using a single‐group model since the multiple‐group model accounts for distributional differences in the individual groups and also possible differential item functioning in the groups. If there are differences between the groups and this is not taken into account, there will exist bias when estimating the item parameters and thus bias when estimating the overall reliability coefficient. Third, with the multiple‐group model, the groupwise and overall reliability coefficients are estimated jointly which provides additional tools to, for example, test the equality of the reliability coefficients in the different groups.

The purpose of this paper is to introduce how group‐specific and overall reliability coefficients for sum scores and maximum likelihood ability estimates can be estimated with unidimensional multiple‐group IRT models for binary and ordinal data. We derive the asymptotic variance of the estimators and outline how to estimate confidence intervals for the reliability coefficients using the asymptotic variance. In addition to the case of known group memberships, we outline how reliability coefficients can be estimated from mixture IRT models (De Ayala & Santiago, 2017; Rost, 1991) where the group memberships are unknown. We exemplify our results by illustrating how the reliability coefficients for scores from the Montreal Cognitive Assessment (MoCA) (Nasreddine et al., 2005) differ between individuals with two different educational levels. The accuracy of the asymptotic variance and the estimated confidence intervals are evaluated using simulated data in the case of known group memberships. To allow implementation of the proposed method, we provide the code as supplementary material.

## 2 IRT with multiple groups

IRT models define a probabilistic relationship between an unobservable continuous latent variable and the observed categorical variables (De Ayala, 2009). IRT models are commonly used in the analysis of scales and tests, with the aim of inferring a latent construct at the level of individuals, evaluating the measurement properties of scales and tests, and inferring population characteristics such as mean differences between groups or the relationship between covariates and a latent construct. What defines a particular IRT model is the probability, conditional on the latent variable, of observing each category of the observed variable. Depending on the model specified, IRT can be used with binary, ordinal or nominal observed variables. In this study we consider the graded response model (Samejima, 1969, GRM) and the generalized partial credit model (Muraki, 1992, GPCM) for ordinal data and the two‐parameter logistic (2PL) model and the three‐parameter logistic (3PL) model for binary data (Birnbaum, 1968); all of these models are commonly used in practice. Besides the functional form of the IRT model, an assumption regarding the marginal distribution of the latent variable is also typically made. For identification of the model parameters, restrictions on the distribution must be imposed. and most commonly the distribution is set to have a mean of 0 and a variance equal to 1.

Individuals taking scales or tests can often be categorized into distinct groups based on their attributes (demographic characteristics, socioeconomic status, or clinical profiles). The hypothesized difference in measurement properties between groups can be assessed and accounted for by multiple‐group IRT models. These models allow for the estimation of the mean and variance in the subgroups, provided that the metric is fixed for one of the groups and that an assumption regarding invariance for some item parameters between pairs of groups is imposed. When this assumption of invariance is fulfilled, the latent distributions of the groups and estimates of the latent variable in the different groups can be compared.

Define *P_jkg_* (*z*; **α** *_jg_*) as the probability, conditional on the latent variable *z*, of obtaining category $k\in \{1,\ldots ,m_{j}\}$ on item *j* in group g∈{1,…,G} where **α** *_jg_* is the vector of item parameters for item *j* in group *g*. For ordinal data with the GRM, the probability is defined as

$$P_{\mathrm{jkg}}\left(z;\;\alpha_{\mathrm{jg}}\right)=P_{j,k,g}^{*}\left(z;\;\alpha_{\mathrm{jg}}\right)‐P_{j,k+1,g}^{*}\left(z;\;\alpha_{\mathrm{jg}}\right),$$

where $P_{j,k\prime ,g}^{*}\left(z;\;\alpha_{\mathrm{jg}}\right)=1/\left(1+\exp\left(‐a_{\mathrm{jg}}z‐b_{j,k\prime ,g}\right)\right)$ for $1<k^{\prime }<m_{j}+1$, $P_{j,1,g}^{*}\left(z;\;\alpha_{\mathrm{jg}}\right)=1$ and $P_{j,m_{j}+1,g}^{*}\left(z;\;\alpha_{\mathrm{jg}}\right)=0$. With the GPCM, the probability is defined by

$$P_{\mathrm{jkg}}\left(z;\;\alpha_{\mathrm{jg}}\right)=\frac{\exp\left[\sum_{v=1}^{k}\left(a_{\mathrm{jg}}z+b_{j,v,g}\right)\right]}{\sum_{c=1}^{m_{j}}\exp\left[\sum_{v=1}^{c}\left(a_{\mathrm{jg}}z+b_{j,v,g}\right)\right]}.$$

With binary data, the above two models are equivalent to the 2PL model. Also with binary data, the 3PL model defines the success or endorsement probability by

$$P_{j2g}\left(z;\;\alpha_{\mathrm{jg}}\right)=c_{\mathrm{jg}}+\frac{1‐c_{\mathrm{jg}}}{1+\exp(‐a_{\mathrm{jg}}z‐b_{\mathrm{jg}})}.$$

A central concept in IRT is the expected item information, which is used to estimate standard errors of estimates of the latent construct and estimate confidence intervals for the latent construct. It is also used in the calculation of the reliability of maximum likelihood estimates (MLEs) in a population. For a general IRT model, the expected item information is equal to (Magis, 2015)

$$I_{\mathrm{jg}}\left(z;\;\alpha_{\mathrm{jg}}\right)=\sum_{k=1}^{m_{j}}\left[\frac{{\left(\frac{\partial P_{\mathrm{jkg}}\left(z;\;\alpha_{\mathrm{jg}}\right)}{\partial z}\right)}^{2}}{P_{\mathrm{jkg}}\left(z;\;\alpha_{\mathrm{jg}}\right)}‐\frac{\partial^{z}P_{\mathrm{jkg}}\left(z;\;\alpha_{\mathrm{jg}}\right)}{\partial z^{2}}\right].$$

Let *J* denote the number of items on a test. The test information function is defined as the sum of the item information functions, namely $I_{g}\left(z;\;\alpha_{g}\right)=\sum_{j=1}^{J}I_{\mathrm{jg}}\left(z;\;\alpha_{\mathrm{jg}}\right)$.

Define $y_{n_{g}}=(y_{n_{g}1g},\ldots ,y_{n_{g}Jg})\prime$ as the item response vector for an individual $n_{g}\in \left\{1,\ldots N_{g}\right\}$ in group *g*, **Y** as the matrix of item response vectors in all groups, ϕ as the normal distribution density function, **α** *_g_* as the item parameter vector in group *g*, **α** = (**α**_1_,…, **α**_G′_)′ as the vector of item parameters in all groups, **µ** = (**µ**_1_,…, **µ**_G_)′ as the vector of mean parameters and $\sigma^{2}={\left(\sigma_{1}^{2},\ldots ,\sigma_{G}^{2}\right)}^{\prime }$ as the vector of variance parameters. We assume independence of the item responses conditional on the latent variable *z*, such that $P(y_{n_{g}}|z;\;\alpha_{g})=\prod_{j=1}^{J}P_{jy_{n_{g}jg}g}(z;\;\alpha_{\mathrm{jg}})$. Then we obtain the marginal likelihood function

$$L\left(\alpha ,\mu ,\sigma^{2}|Y\right)=\prod_{g=1}^{G}\prod_{n_{g}=1}^{N_{g}}\int P\left(y_{n_{g}}|z;\;\alpha_{g}\right)\Phi \left(z;\;\mu_{g},\sigma_{g}^{2}\right)dz,$$

which can be maximized with an EM algorithm (Bock & Aitkin, 1981) to obtain the MLEs of the unknown parameters.

## 3 Estimating reliability coefficients with multiple‐group IRT

After estimating the parameters of an IRT model, it is often desirable to draw inference with respect to the underlying construct that the test items are meant to measure. Hence, the individual latent constructs need to be estimated from the observed item response patterns. One approach, still often used in practice, is to use the sum scores. The sum score for an individual *n_g_* is defined simply as the sum of the individual item scores, namely

$$x_{n_{g}}=\sum_{j=1}^{J}y_{n_{g}jg.}$$

The sum score does not explicitly use the estimated IRT model parameters. However, obtaining an accurate estimate of the reliability of the sum score requires using the estimated model parameters (Green & Yang, 2009; Kim & Feldt, 2010). Another approach commonly used with IRT models to draw inference of the latent construct is to choose a specific estimator and use the estimated model parameters and the response patterns to obtain the estimate of the latent construct. In the literature there are a number of such methods available, such as maximum likelihood (Birnbaum, 1968), the posterior mean (Bock & Aitkin, 1981) and the posterior mode (Samejima, 1969). Here, we focus on the maximum likelihood estimator, ${\hat{z}}_{\mathrm{MLE}}$, which estimates the latent construct for an individual *n_g_* by maximizing the individual likelihood function with respect to *z*. The likelihood is

$$L_{n_{g}}(z|y_{n_{g}};\;\alpha_{g})=\prod_{j=1}^{J}P_{jy_{n_{g}jg}g}(z;\;\alpha_{\mathrm{jg}}),$$

which is maximized with a numerical optimization routine such as the Newton–Raphson method. The variance of the maximum likelihood estimator of the latent construct, as the number of items *J* tends to infinity, is equal to the inverse of the information function and is hence given by

(1) $$Var\left({\hat{z}}_{\mathrm{MLE}}|z\right)=\frac{1}{I_{g}\left(z;\;\alpha_{g}\right)}.$$

For a fixed number of items, the expression in equation (1) is an approximation to the variance. It is possible to improve the approximation via methods discussed in Lord (1983), but for simplicity we will not pursue these in the present paper.

When using either the sum score or the maximum likelihood estimator to draw inference of a latent construct, it is desirable to know the reliability of the scores in a population in order to provide validity evidence of uses of the scores. We can also note that besides their utility in estimating a latent construct, sum scores or MLEs from scales or tests are also often used as the dependent variable or explanatory variable in regression models or structural equation models. Correct application of these models often requires the specification of the reliability of the variable used (Gleser, 1992).

However, the reliabilities of the sum scores and the MLEs are not equal and can vary for different groups. Thankfully we can estimate the reliability of both with the estimated IRT model parameters, as discussed, for example, Green and Yang (2009) and Kim and Feldt (2010) for sum scores and in Nicewander and Thomasson (1999), Cheng et al. (2012) and Kim (2012) for the maximum likelihood estimator and other IRT score estimators. We now proceed to define the reliability of sum scores and MLEs with multiple‐group IRT models, which generalizes previously defined reliability coefficients for such scores to the case of multiple‐group models.

### 3.1 Reliability of sum scores

Let the score of category $k\in \{1,\ldots ,m_{j}\}$ for item g∈{1,…,J} be *W_jk_* and let the sum scores be *x_i_*, i∈{0,…,K}. The reliability of the sum scores in a group *g* can be calculated from the regular definition of the reliability in terms of the ratio of the true score variance to the observed score variance, that is,

$$\rho_{X_{g},X_{g}^{\prime }}=\frac{\sigma_{T_{g}}^{2}}{\sigma_{X_{g}}^{2}}=1‐\frac{\sigma_{e_{g}}^{2}}{\sigma_{X_{g}}^{2}}.$$

For IRT models we have, for each group *g* (Kim & Feldt, 2010),

$$\sigma_{e_{g}}^{2}\left(\alpha ,\mu_{g},\sigma_{g}^{2}\right)=\int \sigma_{e_{g}|z}^{2}\left(\alpha_{g}\right)\Phi \left(z;\;\mu_{g},\sigma_{g}^{2}\right)dz,$$

where

$$\sigma_{e_{g}|z}^{2}\left(\alpha_{g}\right)=\sum_{j=1}^{J}\left[\sum_{k=1}^{m_{j}}P_{\mathrm{jkg}}\left(z;\;\alpha_{\mathrm{gj}}\right)W_{\mathrm{kj}}^{2}‐{\left(\sum_{k=1}^{m_{j}}P_{\mathrm{jkg}}\left(z;\;\alpha_{\mathrm{gj}}\right)W_{\mathrm{jk}}\right)}^{2}\right],$$

and

$$\sigma_{X_{g}}^{2}=\sum_{i=0}^{K}r_{\mathrm{ig}}\left(\alpha_{g},\mu_{g},\sigma_{g}^{2}\right)x_{i}^{2}‐{\left(\sum_{i=0}^{K}r_{\mathrm{ig}}\left(\alpha_{g},\mu_{g},\sigma_{g}^{2}\right)x_{i}\right)}^{2},$$

with $r_{\mathrm{ig}}\left(\alpha_{g},\mu_{g},\sigma_{g}^{2}\right)=\int r_{\mathrm{ig}}\left(z;\;\alpha_{g}\right)\Phi \left(z;\;\mu_{g},\sigma_{g}^{2}\right)dz$. Let $p=(p_{1},\ldots \;p_{G})\prime$ be the vector of proportions of members in each group, treated in a multiple‐group model as fixed and known and treated as unknown parameters in a mixture IRT model. For all groups, we have

$$\rho_{X,X^{\prime }}=1‐\frac{\sigma_{e}^{2}}{\sigma_{X}^{2}},$$

where

$$\sigma_{e}^{2}=\sum_{g=1}^{G}p_{g}\int \sigma_{e_{g}|z}^{2}\left(\alpha_{g}\right)\Phi \left(z;\;\mu_{g},\sigma_{g}^{2}\right)dz,$$

and

$$\sigma_{X}^{2}=\sum_{i=0}^{K}r_{i}\left(\alpha ,p,\mu ,\sigma^{2}\right)x_{i}^{2}‐{\left(\sum_{i=0}^{K}r_{i}\left(\alpha ,p,\mu ,\sigma^{2}\right)x_{i}\right)}^{2},$$

where $r_{i}\left(\alpha ,p,\mu ,\sigma^{2}\right)=\sum_{g=1}^{G}p_{g}\int r_{\mathrm{ig}}\left(z;\;\alpha_{g}\right)\Phi \left(z;\;\mu_{g},\sigma_{g}^{2}\right)dz$. The *r_ig_* (*z*; **α** *_g_*) are the sum score probabilities for group *g* for a given value of *z* which are calculated using a recursive algorithm (Lord & Wingersky, 1984; Thissen, Pommerich, Billeaud, & Williams, 1995). In brief, the algorithm computes the probabilities of each sum score when adding one additional item until all the items have been considered. We obtain the following equation for the sum score probabilities when including *J* ^*^ items:

$$r_{\mathrm{ig}}^{J^{*}}\left(z;\;\alpha_{g}\right)=\sum_{i\prime =0}^{\sum_{j=1}^{J^{*}‐1}W_{\mathrm{jk}}}r_{i\prime g}^{J^{*}‐1}\left(z;\;\alpha_{g}\right)\left(\sum_{k=1}^{m_{j}}P_{J^{*}kg}\left(z;\;\alpha_{g}\right)1\left(k+i\prime ‐1=i\right)\right),$$

where $r_{i\prime g}^{J^{*}‐1}\left(z;\;\alpha_{g}\right)$ are the sum score probabilities for scores $i\prime \in \left\{0,\ldots ,\sum_{j=1}^{J^{*}‐1}W_{\mathrm{jk}}\right\}$, that is, when considering only the *J* ^*^−1 first items. These probabilities are sequentially calculated starting from the first item, where the sum score probabilities are simply equal to the item characteristic function for each category, that is,

$$r_{i\prime g}^{1}\left(z;\;\alpha_{g}\right)=P_{1(i\prime +1)g}(z;\;\alpha_{g}).$$

The integrals in the above equations do not have explicit solutions and need to be approximated. Here, we will use Gauss–Hermite quadrature with nodes *z_l_* and weights *w_l_*, l∈{1,…,L}, for this purpose. For group *g*, let $z_{\mathrm{lg}}=\sqrt{2}\sigma_{g}z_{l}+\mu_{g}$ be the *l*th quadrature point. We then obtain the estimator of the sum score reliability in group *g* as

(2) $${\hat{\rho }}_{X_{g},X_{g^{\prime }}}\left({\hat{\alpha }}_{g},{\hat{\mu }}_{g},{\hat{\sigma }}_{g}^{2}\right)=1‐\frac{\sum_{l=1}^{L}\sigma_{e_{g}}^{2}\left({\hat{\alpha }}_{g}\right){|}^{z={\hat{z}}_{\mathrm{lg}}}w_{l}}{\sum_{i=0}^{K}r_{\mathrm{ig}}\left({\hat{\alpha }}_{g},{\hat{\mu }}_{g},{\hat{\sigma }}_{g}^{2}\right)x_{i}^{2}‐{\left[\sum_{i=0}^{K}r_{\mathrm{ig}}\left({\hat{\alpha }}_{g},{\hat{\mu }}_{g},{\hat{\sigma }}_{g}^{2}\right)x_{i}\right]}^{2}},$$

where $r_{\mathrm{ig}}\left({\hat{\alpha }}_{g},{\hat{\mu }}_{g},{\hat{\sigma }}_{g}^{2}\right)=\sum_{l=1}^{L}r_{\mathrm{ig}}\left({\hat{z}}_{\mathrm{lg}};\;{\hat{\alpha }}_{g}\right)w_{l}$. Similarly, we obtain the estimator of the reliability of the sum scores from all groups,

(3) $${\hat{\rho }}_{X,X^{\prime }}\left(\hat{\alpha },\hat{p},\hat{\mu },{\hat{\sigma }}^{2}\right)=1‐\frac{\sum_{g=1}^{G}{\hat{p}}_{g}\sum_{l=1}^{L}\sigma_{e_{g}|z}^{2}\left({\hat{\alpha }}_{g}\right){|}^{z={\hat{z}}_{\mathrm{lg}}}w_{l}}{\sum_{i=0}^{K}r_{i}\left(\hat{\alpha },\hat{p},\hat{\mu },{\hat{\sigma }}^{2}\right)x_{i}^{2}‐{\left[\sum_{i=0}^{K}r_{i}\left(\hat{\alpha },\hat{p},\hat{\mu },{\hat{\sigma }}^{2}\right)x_{i}\right]}^{2}},$$

where $r_{i}\left(\hat{\alpha },\hat{p},\hat{\mu },{\hat{\sigma }}^{2}\right)=\sum_{g=1}^{G}{\hat{p}}_{g}\sum_{l=1}^{L}r_{\mathrm{ig}}\left({\hat{z}}_{\mathrm{lg}};\;{\hat{\alpha }}_{g}\right)w_{l}$.

### 3.2 Reliability of maximum likelihood ability estimates

The reliability of the maximum likelihood ability estimates in a population can be found by considering the ratio of the latent distribution variance to the total variance, where the total variance is equal to the sum of the latent distribution variance and the error variance (Cheng et al., 2012). When computing the error variance, we will integrate the expression given in equation (1) over the latent distribution in each group. The approach we use gives the reliability coefficient of the maximum likelihood ability estimates as the number of items tends to infinity. For a detailed discussion of reliability coefficients for different types of ability estimators, see S. Kim (2012). For a group *g* with latent distribution variance $\sigma_{g}^{2}$ we thus have that the reliability of the MLEs is

$$\rho_{\Theta_{g}}\left(\alpha_{g},\mu_{g},\sigma_{g}^{2}\right)=\frac{\sigma_{g}^{2}}{\sigma_{g}^{2}+\int \frac{1}{I_{g}\left(z;\;\alpha_{g}\right)}\Phi \left(z;\;\mu_{g},\sigma_{g}^{2}\right)dz},$$

where **α** *_g_*, µ*_g_* and $\sigma_{g}^{2}$ are the unknown parameters which may potentially differ between groups. We now consider the reliability of the maximum likelihood ability estimates for all groups, defined as

$$\rho_{\Theta }\left(\alpha ,p,\mu ,\sigma^{2}\right)=\frac{\sigma^{2}}{\sigma^{2}+\sum_{g=1}^{G}\;p_{g}\int \frac{1}{I_{g}\left(z;\;\alpha_{g}\right)}\Phi \left(z;\;\mu_{g},\sigma_{g}^{2}\right)dz},$$

where $\sigma^{2}=\sum_{g=1}^{G}\;p_{g}\left({\left(\mu_{g}‐\mu \right)}^{2}+\sigma_{g}^{2}\right)$, with $\mu =\sum_{g=1}^{G}\;p_{g}\mu_{g}$, that is, µ and σ^2^ are the mean and variance of the mixture distribution for all groups, respectively (McLachlan & Peel, 2000). Note that we have defined $p_{1}=1‐\sum_{g=2}^{G}\;p_{g}$, and to identify the unknown parameters we have imposed the restrictions µ_1_ = 0 and $\sigma_{1}^{2}=1$.

We again approximate the required integral with Gauss–Hermite quadrature and the estimator of the reliability of the maximum likelihood ability estimates in group *g* is then a function of the item and distribution parameter estimators, that is,

(4) $${\hat{\rho }}_{\Theta_{g}}\left({\hat{\alpha }}_{g},{\hat{\mu }}_{g},{\hat{\sigma }}_{g}^{2}\right)=\frac{{\hat{\sigma }}_{g}^{2}}{{\hat{\sigma }}_{g}^{2}+\sum_{l=1}^{L}\frac{1}{I_{g}\left({\hat{z}}_{\mathrm{lg}},{\hat{\alpha }}_{g}\right)}w_{l}}.$$

Similarly, the estimator of the reliability of the MLEs for all groups is

(5) $${\hat{\rho }}_{\Theta }(\hat{\alpha },\hat{p},\hat{\mu },{\hat{\sigma }}_{2})=\frac{{\hat{\sigma }}^{2}}{{\hat{\sigma }}^{2}+\sum_{g=1}^{G}{\hat{p}}_{g}\sum_{l=1}^{L}\frac{1}{I_{g}({\hat{z}}_{\mathrm{lg}},{\hat{\alpha }}_{g})}w_{l}}.$$

### 3.3 Confidence interval estimation

The large‐sample variances of the estimators in equations ((2), (3), (4), (5)) can be derived with standard methods from asymptotic theory (Ferguson, 1996). For an estimator $\hat{\rho }$ that is a function of a parameter estimator $\hat{\xi }$ with asymptotic covariance matrix $\sum_{\hat{\xi }}$, we have that the asymptotic covariance matrix for $\hat{\rho }$ can be approximated by the delta method with

(6) $$\sum_{\hat{\rho }}\approx \frac{\partial \hat{\rho }}{\partial \hat{\xi }}\sum_{\hat{\xi }}{\left(\frac{\partial \hat{\rho }}{\partial \hat{\xi }}\right)}^{'}$$

In a particular instance $\hat{\xi }$ is replaced by the IRT model parameter estimates to obtain the estimated covariance matrix. The variance of the sum score reliability estimator was presented in Andersson and Xin (2018) for single‐group IRT models with the 3PL model and GPCM. For the multiple‐group reliability coefficients defined in this paper, we need to account for the additional parameters *p_g_*, µ*_g_* and $\sigma_{g}^{2}$, compute the derivatives pertaining to the reliability coefficient for the maximum likelihood reliability estimates and also provide support for the GRM. The required derivatives for this are given in the Appendix S1; they are partly based on the derivatives from Andersson and Xin (2020). We verified the analytical derivatives by comparing them with numerical derivatives. With the asymptotic covariance matrix, we can estimate approximate confidence intervals for a reliability coefficient $\rho_{q}\in \rho$ from the estimated standard error $\hat{\mathrm{SE}}\left({\hat{\rho }}_{q}\right)=\sqrt{\hat{V}ar\left({\hat{\rho }}_{q}\right)}$. For example, a 95% confidence interval is estimated with ${\hat{\rho }}_{q}^{\mathrm{obs}}\pm z_{0.975}\times \hat{\mathrm{SE}}\left({\hat{\rho }}_{q}\right)$, where *z*_0.975_ denotes the 0.975 quantile of the standard normal distribution. We note here that it is also possible to convert the item parameter estimates from different groups onto a common metric and utilize the expressions in, for example, Cheng et al. (2012) and S. Kim and Feldt (2010) to compute the groupwise reliability coefficients defined in equations (2) and (4). Such conversions would give the same estimates but would require different derivations for the standard error estimation than those presented here.

## 4 Empirical example: Estimating reliabilities of moca scores in a large hong kong Sample

In this empirical example, we used data from 1,873 older persons with and without formal education in Hong Kong to demonstrate the procedure of estimating group‐specific and overall reliability coefficients of the MoCA scores with multiple‐group IRT models. The MoCA is a widely used tool for screening cognitive impairment and dementia (Nasreddine et al., 2005). When applying the MoCA for cognitive assessment, a common concern is the effect of educational level on the test scores. Substantially different cut‐off values for mild cognitive impairment and dementia have been proposed for people with different educational levels (Balsis, Choudhury, Geraci, Benge, & Patrick, 2018). An earlier study applied multiple‐group IRT analysis to the MoCA in a low‐education older population in Hong Kong and found that item characteristics differed between older persons with and without formal education (Luo, Andersson, Tang, & Wong, 2020) by employing a model selection procedure with the Bayesian information criterion. Using the same data and model, we extended the earlier analysis by including estimation of the reliability coefficients using multiple‐group IRT models. In this sample, 45% of the respondents had no formal education. The earlier analysis utilized the GRM and identified three items that functioned differently with respect to education level: Cube, clock number and clock hand. The selected model had an root mean squared error of approximation of 0.042 (95% CI 0.038–0.048) and a standardized root mean squared residual of 0.055 and 0.054, indicating good model fit Maydeu‐Olivares, 2013; Maydeu‐Olivares & Joe, 2014).

Based on the selected model from the earlier study, we then estimated the sum score reliability and the maximum likelihood estimator reliability for each group (from equations (2) and (4)) and the total sample (from equations (3) and (5)). The point estimates and associated confidence intervals are presented in Table 1. The mean and variance of the ‘some formal education’ group was set to 0 and 1, respectively, to identify the model parameters. The reliability estimates are higher in the ‘no formal education’ group. The test information functions for both groups are plotted in Figure A1 in Appendix S2, showing that the measurement precision differs between the groups across the latent scale. The three items expressing differential item functioning with respect to the educational level attained have an overall higher measurement precision for the ‘no formal education group’ at the middle of the latent scale. These findings, along with the differing distribution parameter estimates in the groups, explain why the measurement precision is lower in the ‘some formal education’ group than in the ‘no formal education’ group.

**Table 1:** Estimated mean cognitive performance ($\hat{\mu }$), variance of cognitive performance (${\hat{\sigma }}^{2}$), sum score reliability and maximum likelihood estimate (MLE) reliability in two education levels and overall, with standard errors in parentheses

Education level	$\hat{\mu }$	${\hat{\sigma }}^{2}$	Sum score reliability	MLE reliability
Some formal education	0 (−)	1 (−)	0.736 (0.012)	0.760 (0.009)
No formal education	−1.081 (0.063)	1.096 (0.103)	0.785 (0.010)	0.819 (0.008)
All education levels	−0.486 (0.028)	1.332 (0.066)	0.806 (0.007)	0.825 (0.005)

## 5 Monte Carlo simulations

We conducted a Monte Carlo simulation study to verify the derivations provided, evaluate the estimation properties and assess the finite‐sample properties of the confidence intervals. The IRT models were estimated with the R (R Core Team, 2020) package *mirt* (Chalmers, 2012), using marginal maximum likelihood with the EM algorithm. The asymptotic covariance matrix was estimated with the sandwich estimator (Yuan, Cheng, & Patton, 2014). Newly written R code was used to estimate the reliability coefficients and confidence intervals. The code can be found in the online supplementary material. In the simulations, we evaluated the bias of the reliability estimators and the bias of the confidence interval estimators. To compute the bias, we defined the true reliability coefficients by the values obtained from plugging in the true distribution parameters and item parameters via equations (2)–(5) and compared these to the average of the estimates in the Monte Carlo simulation. We also evaluated the coverage rate of 95% confidence intervals estimated with the standard errors. To evaluate the estimation efficiency of the multiple‐group model reliability coefficients, estimators based on single‐group and multiple‐group models were compared by considering the relative efficiency. The relative efficiency was computed using the ratio of the estimated mean squared errors (variance plus the squared bias) of the estimators.

The simulation study was designed to mimic the example with the MoCA. We simulated item parameters based on the estimated two‐group GRM to simulate item response data with 14 and 28 items in two groups, where the weights were consistent with the empirical example (45% and 55%) and where three out of 14 items and six out of 28 items had differential item functioning in the respective settings. For each replication, four IRT models were estimated: three single‐group IRT models for the data in each of the two individual groups and the total sample, and one multiple‐group model where the invariance constraints were consistent with the empirical example. The IRT models were estimated using marginal maximum likelihood, and then the sum score and MLE reliability coefficients based on the single‐group models and the multiple‐group model were estimated. We considered sample sizes 1,000, 2,000 and 4,000, with 5,000 replications in each setting.

The simulation results showed that the non‐convergence rates with 14 items were 6%, 0.36% and 0%, and with 28 items 9.74%, 0.6%, and 0%, for sample sizes 1,000, 2,000 and 4,000, respectively. The results for the bias are given in Table 2, showing that the estimators are essentially unbiased. The empirical coverage rates of 95% confidence intervals are given in Table 3. The results indicate that all reliability coefficient confidence intervals have coverage close to the nominal level with all sample sizes, with exception of the confidence intervals for the MLE reliability coefficient which are slightly below the nominal level with sample size 1,000 for some settings. We also investigated the relative efficiency of MLE and sum score reliability estimators from single‐group models relative to estimators from multiple‐group models and found improved efficiencies in estimators from multiple‐group models across all settings (Table 4).

**Table 2:** Bias for the sum score and MLE reliability estimators, with the 14‐ and 28‐item graded response models

*N*	Group 1	Group 2	All groups	Group 1	Group 2	All groups
	14 items			28 items		
Bias of sum score reliability estimators						
1,000	−0.0001	−0.0009	0.0000	0.0004	−0.0004	0.0001
2,000	−0.0000	−0.0004	0.0001	0.0003	−0.0001	0.0001
4,000	0.0001	−0.0002	0.0000	0.0003	−0.0001	0.0000
Bias of MLE reliability estimators						
1,000	0.0009	0.0003	0.0010	0.0008	0.0003	0.0006
2,000	0.0004	0.0002	0.0005	0.0005	0.0002	0.0003
4,000	0.0004	0.0001	0.0003	0.0004	0.0000	0.0001

**Table 3:** Empirical coverage rates (%) of 95% confidence intervals for the sum score and MLE reliabilities, with bold font indicating that the coverage rate is statistically significantly different from 95%

*N*	Group 1	Group 2	All groups	Group 1	Group 2	All groups
	14 items			28 items		
Sum score reliability estimators						
1,000	95.00	94.51	94.94	94.48	94.68	94.62
2,000	94.86	95.04	95.14	95.01	94.71	95.15
4,000	95.28	94.72	95.34	94.86	95.12	94.94
MLE reliability estimators						
1,000	94.62	94.53	**94.23**	**93.82**	**94.17**	**94.04**
2,000	94.72	94.70	94.44	94.71	94.65	94.83
4,000	95.18	94.72	95.14	94.70	94.98	94.64

**Table 4:** Relative efficiency of sum score and MLE reliability estimators from single‐group models relative to estimators from multiple‐group models

*N*	Group 1	Group 2	All groups	Group 1	Group 2	All groups
	14 items			28 items		
Sum score reliability estimators						
1,000	1.12	1.14	1.02	1.06	1.07	1.06
2,000	1.14	1.13	1.04	1.07	1.06	1.14
4,000	1.13	1.11	1.08	1.09	1.08	1.36
MLE reliability estimators						
1,000	1.05	1.05	1.11	1.02	1.04	1.10
2,000	1.05	1.04	1.24	1.02	1.03	1.19
4,000	1.05	1.05	1.49	1.03	1.03	1.33

## 6 Discussion

In this paper we introduced reliability coefficients for sum scores and maximum likelihood ability estimates based on multiple‐group IRT models. We derived the asymptotic variance of the reliability coefficient estimators and evaluated the finite‐sample properties of the estimators and confidence intervals with simulations. Our results show that the reliability coefficient estimators are largely unbiased and that the confidence intervals have correct empirical coverage rates. With the results provided, applied researchers can estimate the groupwise and overall reliability of sum scores and MLEs directly from the estimated multiple‐group IRT model parameters. This enables a better evaluation of the measurement properties of scale scores in diverse groups.

An alternative to estimating the reliability of sum scores and maximum likelihood ability estimates with a multiple‐group model is to fit individual models for each group and estimate the reliability separately in each group. However, such a procedure does not encompass a study of measurement invariance across groups and hence does not enable a common interpretation of the resulting scores across the groups. In addition, the estimation accuracy and precision are negatively impacted since only the observations in each individual group are used instead of the entire data. In the present study, the estimation efficiency decreased markedly when using single‐group models compared to multiple‐group modelling. Furthermore, separate estimation in each group does not provide a method to estimate the reliability of scores for all groups combined. If instead using a joint single‐group model to estimate the reliability for all groups combined, the potential differences in the item parameters across groups and in the latent distribution parameters are not taken into account. If the parameters differ between the groups, the estimation of the reliability with a single‐group model may be biased. Such bias impacted the estimation performance negatively for the overall reliability coefficients based on the single‐group model in the simulation study. Hence we view utilizing the multiple‐group model as a major advantage when estimating the reliability.

We offer some suggestions for the practical use of our results. Since the reliability coefficients are defined by a multiple‐group IRT model, it is essential to establish appropriate model fit before interpreting the results from the reliability coefficients presented in this paper. If the model does not fit well, the subsequent reliability coefficient estimates can be biased. The results of the simulation study provide support for the use of the estimators and confidence intervals with a sample size per group as small as 500, although the confidence intervals for the maximum likelihood ability estimate reliability had empirical coverage rates that were lower than the nominal level with sample size 500. The sample size requirements for multiple‐group model estimation vary with the characteristics of the groups and the number and types of items. Generally speaking, larger differences between groups require larger sample sizes and more items with more categories also require larger sample sizes.

There are some limitations to the present study. First, the derivations presented assume a normal distribution as the latent variable distribution in each group, which is somewhat restrictive. However, we also note that a multiple‐group model can be a tool to model non‐normality in IRT since multiple‐group models assume that the unconditional latent distribution is a mixture of multiple‐groupwise normal distributions, which can be highly non‐normal. Second, we only considered a selection of IRT models in our study. Although the 2PL model, 3PL model, GPCM and GRM are the most commonly used models, there are a large number of additional models available in the literature. However, note that the derivations presented apply to general unidimensional IRT models, which means that the results can be easily adapted to additional models. Third, we note that while we presented how to estimate confidence intervals using a normal approximation, this procedure can be extended and modified further. The confidence intervals we presented are symmetric intervals that do not take into consideration the restricted range of the reliability coefficients. Instead of a normal approximation, we can utilize alternative methods (Cox & Ma, 1995) to account for the restricted range of the reliability coefficients and obtain confidence intervals that are more informative when the estimates are close to the boundary of the parameter space. Fourth, we did not consider the impact of item parameter estimation on the reliability of the maximum likelihood ability estimates, as done via multiple imputation in Yang, Hansen, and Cai (2012). Lastly, the maximum likelihood ability estimator and the subsequent MLE reliability coefficients may be biased when the number of items is not sufficiently large. However, we did not consider finite‐item adjustments to the MLE reliability coefficients. By utilizing expressions from Lord (1983), it is possible to improve estimation accuracy of the multiple‐group maximum likelihood reliability estimators when the number of items is small.

We also note a further application of our results that may be useful in practice. The asymptotic covariance matrix for the reliability coefficient estimator can be used to test hypotheses regarding the reliability coefficients by utilizing a Wald test (Wald, 1943). For example, we can test the equality of the reliability of sum scores between groups or we can test whether a reliability coefficient is statistically significantly larger or smaller than a particular value. Such tests can be directly applied with the derivations presented in this paper.

Future extensions to the methods presented here are possible. It is possible to extend the approach presented to multidimensional IRT models, and obtain more accurate estimation of the score reliability when a unidimensional model is not tenable. In addition, it is possible to consider the reliability of other IRT score estimators such as the posterior mean and the posterior mode when using multiple‐group models.

## Author contributions

**Kseniia Marcq** (Software; Writing – review & editing) **Hao Luo** (Data curation; Formal analysis; Methodology; Resources; Writing – review & editing) **Björn Andersson** (Conceptualization; Formal analysis; Methodology; Software; Writing – original draft).

## Conflicts of interest

All authors declare no conflict of interest.

## Supporting information


**Appendix S1**. Derivatives and table.

**Appendix S2**. R code to implement the methodology.

**Appendix S3**. R code with an example.
