# Supplementary material for: Reliability coefficients for multiple group item response theory models
Source: Br J Math Stat Psychol. 2022 Mar 1;75(2):395–410. doi: 10.1111/bmsp.12269 (PMC9313586; doi:10.1111/bmsp.12269)
Supplement: Supplementary file 1 — Appendix S1. Derivatives and table. [file BMSP-75-395-s002.docx]

**Appendix A**

# Derivatives

We first consider the derivatives of $\rho_{\Theta_{g}}$ and $\rho_{\Theta}$. For group $g$ we have

$$\begin{matrix} \frac{\partial\rho_{\Theta_{g}}}{\partial\boldsymbol{\alpha}_{g}}=\frac{\sigma_{g}^{2}}{\left( \sigma_{g}^{2}+\sum_{l=1}^{L} \frac{1}{I_{g}\left( z_{lg};\boldsymbol{\alpha}_{g} \right)}w_{l} \right)^{2}}\sum_{l=1}^{L} \frac{1}{\left[ I_{g}\left( z_{lg};\boldsymbol{\alpha}_{g} \right) \right]^{2}}\left. \frac{\partial I_{g}\left( z;\boldsymbol{\alpha}_{g} \right)}{\partial\boldsymbol{\alpha}_{g}} \right|^{z=z_{lg}}w_{l}, \end{matrix}$$

where $\frac{\partial I_{g}\left( z;\boldsymbol{\alpha}_{g} \right)}{\partial\boldsymbol{\alpha}_{g}}$ has entries

$$\begin{matrix} \frac{\partial I_{jg}\left( z;\boldsymbol{\alpha}_{g} \right)}{\partial\boldsymbol{\alpha}_{jg}}=\sum_{k=1}^{m_{j}} \left[ 2\frac{\partial P_{jkg}\left( z;\boldsymbol{\alpha}_{jg} \right)}{\partial z}\frac{\frac{\partial^{2}P_{jkg}\left( z;\boldsymbol{\alpha}_{jg} \right)}{\partial z\partial\boldsymbol{\alpha}_{jg}}}{P_{jkg}\left( z;\boldsymbol{\alpha}_{jg} \right)}-\frac{\left( \frac{\partial P_{jkg}\left( z;\boldsymbol{\alpha}_{jg} \right)}{\partial z} \right)^{2}\frac{\partial P_{jkg}\left( z;\boldsymbol{\alpha}_{jg} \right)}{\partial\boldsymbol{\alpha}_{jg}}}{\left( P_{jkg}\left( z;\boldsymbol{\alpha}_{jg} \right) \right)^{2}}-\frac{\partial^{3}P_{jkg}\left( z;\boldsymbol{\alpha}_{jg} \right)}{\partial z^{2}\partial\boldsymbol{\alpha}_{jg}} \right]. \end{matrix}$$

We also have

$$\begin{matrix} \frac{\partial\rho_{\Theta_{g}}}{\partial\mu_{g}}= & \frac{\sigma_{g}^{2}}{\left( \sigma_{g}^{2}+\sum_{l=1}^{L} \frac{1}{I_{g}\left( z_{lg};\boldsymbol{\alpha}_{g} \right)}w_{l} \right)^{2}}\sum_{l=1}^{L} \frac{1}{\left[ I_{g}\left( z;\boldsymbol{\alpha}_{g} \right) \right]^{2}}\left. \frac{\partial I_{g}\left( z;\boldsymbol{\alpha}_{g} \right)}{\partial z} \right|^{z=z_{lg}}w_{l} \end{matrix}$$

where

$$\begin{matrix} \frac{\partial I_{g}\left( z;\boldsymbol{\alpha}_{jg} \right)}{\partial z}=\sum_{j=1}^{J} \sum_{k=1}^{m_{j}} \left[ 2\frac{\partial P_{jkg}\left( z;\boldsymbol{\alpha}_{jg} \right)}{\partial z}\frac{\frac{\partial^{2}P_{jkg}\left( z;\boldsymbol{\alpha}_{jg} \right)}{\partial z^{2}}}{P_{jkg}\left( z;\boldsymbol{\alpha}_{jg} \right)}-\frac{\left( \frac{\partial P_{jkg}\left( z;\boldsymbol{\alpha}_{jg} \right)}{\partial z} \right)^{3}}{\left( P_{jkg}\left( z;\boldsymbol{\alpha}_{jg} \right) \right)^{2}}-\frac{\partial^{3}P_{jkg}\left( z;\boldsymbol{\alpha}_{jg} \right)}{\partial z^{3}} \right], \end{matrix}$$

and

$$\begin{matrix} \frac{\partial\rho_{\Theta_{g}}}{\partial\sigma_{g}^{2}}= & \frac{1}{\sigma_{g}^{2}+\sum_{l=1}^{L} \frac{1}{I_{g}\left( z_{lg};\boldsymbol{\alpha}_{g} \right)}w_{l}}-\frac{\sigma_{g}^{2}}{\left( \sigma_{g}^{2}+\sum_{l=1}^{L} \frac{1}{I_{g}\left( z_{lg};\boldsymbol{\alpha}_{g} \right)}w_{l} \right)^{2}} \\ & +\frac{\sigma_{g}^{2}}{\left( \sigma_{g}^{2}+\sum_{l=1}^{L} \frac{1}{I_{g}\left( z_{lg};\boldsymbol{\alpha}_{g} \right)}w_{l} \right)^{2}}\sum_{l=1}^{L} \frac{1}{\left[ I_{g}\left( z;\boldsymbol{\alpha}_{g} \right) \right]^{2}}\left. \frac{\partial I_{g}\left( z;\boldsymbol{\alpha}_{g} \right)}{\partial z} \right|^{z=z_{lg}}\frac{\partial z_{lg}}{\partial\sigma_{g}^{2}}w_{l}, \end{matrix}$$

where $\frac{\partial z_{lg}}{\partial\sigma_{g}^{2}}=\frac{z_{l}}{\sqrt{2}\sigma_{g}}$. The derivatives $\frac{\partial P_{jkg}\left( z;\boldsymbol{\alpha}_{jg} \right)}{\partial z}$, $\frac{\partial^{2}P_{jkg}\left( z;\boldsymbol{\alpha}_{jg} \right)}{\partial z^{2}}$, $\frac{\partial^{3}P_{jkg}\left( z;\boldsymbol{\alpha}_{jg} \right)}{\partial z^{3}}$, $\frac{\partial P_{jkg}\left( z;\boldsymbol{\alpha}_{jg} \right)}{\partial\boldsymbol{\alpha}_{jg}}$, $\frac{\partial^{2}P_{jkg}\left( z;\boldsymbol{\alpha}_{jg} \right)}{\partial z\partial\boldsymbol{\alpha}_{jg}}$ and $\frac{\partial^{3}P_{jkg}\left( z;\boldsymbol{\alpha}_{jg} \right)}{\partial z^{2}\partial\boldsymbol{\alpha}_{jg}}$ for the GPCM, GRM and 3-PL model were given in Andersson and Xin (2021). For the overall reliability, we have, for $g\in\{1,\ldots,G\}$,

$$\begin{matrix} \frac{\partial\rho_{\Theta}}{\partial\boldsymbol{\alpha}_{g}}= & \frac{\sigma^{2}}{\left( \sigma^{2}+\sum_{g=1}^{G} p_{g}\sum_{l=1}^{L} \frac{1}{I_{g}\left( z_{lg};\boldsymbol{\alpha}_{g} \right)}w_{l} \right)^{2}}p_{g}\sum_{l=1}^{L} \frac{1}{\left[ I_{g}\left( z_{lg};\boldsymbol{\alpha}_{g} \right) \right]^{2}}\frac{\partial I_{g}\left( z;\boldsymbol{\alpha}_{g} \right)}{\partial\boldsymbol{\alpha}_{g}}w_{l}, \end{matrix}$$

and, for $\xi\in\{\mu_{g'},\sigma_{g'}^{2}\}$, with $g'\in\{2,\ldots,G\}$,

$$\begin{matrix} \frac{\partial\rho_{\Theta}}{\partial\xi}= & \frac{\frac{\partial\sigma^{2}}{\partial\xi}}{\sigma^{2}+\sum_{g=1}^{G} p_{g}\sum_{l=1}^{L} \frac{1}{I_{g}\left( z_{lg};\boldsymbol{\alpha}_{g} \right)}w_{l}}-\frac{\sigma^{2}}{\left( \sigma^{2}+\sum_{g=1}^{G} p_{g}\sum_{l=1}^{L} \frac{1}{I_{g}\left( z_{lg};\boldsymbol{\alpha}_{g} \right)}w_{l} \right)^{2}}\frac{\partial\sigma^{2}}{\partial\xi} \\ & +\frac{\sigma^{2}}{\left( \sigma^{2}+\sum_{g=1}^{G} p_{g}\sum_{l=1}^{L} \frac{1}{I_{g}\left( z_{lg};\boldsymbol{\alpha}_{g} \right)}w_{l} \right)^{2}}p_{g'}\sum_{l=1}^{L} \frac{1}{\left[ I_{g'}\left( z_{lg'};\boldsymbol{\alpha}_{g'} \right) \right]^{2}}\left. \frac{\partial I_{g'}\left( z;\boldsymbol{\alpha}_{g'} \right)}{\partial z} \right|^{z=z_{lg'}}\frac{\partial z_{lg'}}{\partial\xi}w_{l}, \end{matrix}$$

where $\frac{\partial\sigma^{2}}{\partial\sigma_{g'}^{2}}=p_{g'}$, $\frac{\partial z_{lg}}{\partial\mu_{g'}}=1$ and $\frac{\partial\sigma^{2}}{\partial\mu_{g'}}=-\sum_{g=1}^{G} 2p_{g}\left( \mu_{g}-\mu\right)\frac{\partial\mu}{\partial\mu_{g'}}$, where $\frac{\partial\mu}{\partial\mu_{g'}}=p_{g'}$. We also have $\begin{matrix} \frac{\partial\rho_{\Theta}}{\partial p_{g'}}= & \frac{\frac{\partial\sigma^{2}}{\partial p_{g'}}}{\sigma^{2}+\sum_{g=1}^{G} p_{g}\sum_{l=1}^{L} \frac{1}{I_{g}\left( z_{lg};\boldsymbol{\alpha}_{g} \right)}w_{l}}-\frac{\sigma^{2}}{\left( \sigma^{2}+\sum_{g=1}^{G} p_{g}\sum_{l=1}^{L} \frac{1}{I_{g}\left( z_{lg};\boldsymbol{\alpha}_{g} \right)}w_{l} \right)^{2}}\frac{\partial\sigma^{2}}{\partial p_{g'}} \\ & -\frac{\sigma^{2}}{\left( \sigma^{2}+\sum_{g=1}^{G} p_{g}\sum_{l=1}^{L} \frac{1}{I_{g}\left( z_{lg};\boldsymbol{\alpha}_{g} \right)}w_{l} \right)^{2}}\sum_{g=1}^{G} \frac{\partial p_{g}}{\partial p_{g'}}\sum_{l=1}^{L} \frac{1}{I_{g}\left( z_{lg};\boldsymbol{\alpha}_{g} \right)}w_{l}, \end{matrix}$ where $\begin{matrix} \frac{\partial p_{g}}{\partial p_{g'}}=\left\{ \begin{matrix} -1, & g=1 \\ 1, & g=g' \\ 0, & \text{else} \end{matrix} \right. \end{matrix}$ and $\begin{matrix} \frac{\partial\sigma^{2}}{\partial p_{g'}}=\sum_{g=1}^{G} \left[ \frac{\partial p_{g}}{\partial p_{g'}}\left( \left( \mu_{g}-\mu\right)^{2}+\sigma_{g}^{2} \right)-2p_{g}\left( \mu_{g}-\mu\right)\frac{\partial\mu}{\partial p_{g'}} \right], \end{matrix}$ with $\frac{\partial\mu}{\partial p_{g'}}=\mu_{g'}$.

We now turn to the derivatives of $\rho_{X,X'}$. We first note that

$$\begin{matrix} \frac{\partial\sigma_{e_{g}|z}^{2}}{\partial z}=\sum_{j=1}^{J} \left[ \sum_{k=1}^{m_{j}} \frac{\partial P_{jkg}\left( z;\boldsymbol{\alpha}_{g} \right)}{\partial z}W_{jk}^{2}-2\left( \sum_{k=1}^{m_{j}} P_{jkg}\left( z;\boldsymbol{\alpha}_{g} \right)W_{jk} \right)\sum_{k=1}^{m_{j}} \frac{\partial P_{jkg}\left( z;\boldsymbol{\alpha}_{g} \right)}{\partial z}W_{jk} \right]. \end{matrix}$$

We then have, for $\xi\in\{\boldsymbol{\alpha},\mathbf{p},\boldsymbol{\mu},\boldsymbol{\sigma}^{2}\}$,

$$\begin{matrix} \frac{\partial\rho_{X,X'}}{\partial\xi}=-\frac{\frac{\partial\sigma_{e}^{2}}{\partial\xi}}{\sigma_{X}^{2}}+\frac{\sigma_{e}^{2}}{\sigma_{X}^{4}}\frac{\partial\sigma_{X}^{2}}{\partial\xi}, \end{matrix}$$

where

$$\begin{matrix} \frac{\partial\sigma_{e}^{2}}{\partial\mu_{g}}=p_{g}\sum_{l=1}^{L} \left. \frac{\partial\sigma_{e_{g}|z}^{2}}{\partial z} \right|^{z=z_{lg}}w_{l}, \end{matrix}$$

$$\begin{matrix} \frac{\partial\sigma_{e}^{2}}{\partial\sigma_{g}^{2}}=p_{g}\sum_{l=1}^{L} \left. \frac{\partial\sigma_{e_{g}|z}^{2}}{\partial z} \right|^{z=z_{lg}}\frac{\partial z_{lg}}{\partial\sigma_{g}^{2}}w_{l}, \end{matrix}$$

and

$$\begin{matrix} \frac{\partial\sigma_{e}^{2}}{\partial p_{g'}}=\sum_{g=1}^{G} \frac{\partial p_{g}}{\partial p_{g'}}\sum_{l=1}^{L} \left. \sigma_{e_{g}|z}^{2} \right|^{z=z_{lg}}w_{l}, \end{matrix}$$

and

$$\begin{matrix} \frac{\partial\sigma_{X}^{2}}{\partial\xi}=\sum_{i=0}^{K} \frac{\partial r_{i}}{\partial\xi}x_{i}^{2}-2\left( \sum_{i=0}^{K} r_{i}x_{i} \right)\left( \sum_{i=0}^{K} \frac{\partial r_{i}}{\partial\xi}x_{i} \right), \end{matrix}$$

where

$$\begin{matrix} \frac{\partial r_{i}}{\partial\mu_{g}}=p_{g}\sum_{l=1}^{L} \left. \frac{\partial r_{ig}\left( z;\boldsymbol{\alpha}_{g} \right)}{\partial z} \right|^{z=z_{lg}}w_{l}, \end{matrix}$$

$$\begin{matrix} \frac{\partial r_{i}}{\partial\sigma_{g}^{2}}=p_{g}\sum_{l=1}^{L} \left. \frac{\partial r_{ig}\left( z;\boldsymbol{\alpha}_{g} \right)}{\partial z} \right|^{z=z_{lg}}\frac{\partial z_{lg}}{\partial\sigma_{g}^{2}}w_{l} \end{matrix}$$

and

$$\begin{matrix} \frac{\partial r_{i}}{\partial p_{g'}}=\sum_{g=1}^{G} \frac{\partial p_{g}}{\partial p_{g'}}\sum_{l=1}^{L} r_{ig}\left( z_{lg};\boldsymbol{\alpha}_{g} \right)w_{l}. \end{matrix}$$

To calculate the derivatives of the sum score probabilities, $\frac{\partial r_{ig}\left( z;\boldsymbol{\alpha}_{g} \right)}{\partial z}$, we take the derivative of the probabilities in the recursive algorithm, leading to

$$\begin{matrix} \frac{\partial r_{ig}\left( z;\boldsymbol{\alpha}_{g} \right)}{\partial z}=\sum_{i'=0}^{\sum_{j=1}^{J-1} \left( m_{j}-1 \right)} & \left[ \frac{\partial r_{i'g}^{J-1}\left( z;\boldsymbol{\alpha}_{g} \right)}{\partial z}\left( \sum_{k=1}^{m_{j}} P_{Jkg}\left( z;\boldsymbol{\alpha}_{g} \right)\mathbf{1}\left( k+i'-1=i \right) \right) \right. \\ & \left. +r_{i'g}^{J-1}\left( z;\boldsymbol{\alpha}_{g} \right)\left( \sum_{k=1}^{m_{j}} \frac{\partial P_{Jkg}\left( z;\boldsymbol{\alpha}_{g} \right)}{\partial z}\mathbf{1}\left( k+i'-1=i \right) \right) \right], \end{matrix}$$

where $r_{i'g}^{J-1}\left( z;\boldsymbol{\alpha}_{g} \right)$ are the sum score probabilities when considering only the items up to and including item $J-1$. In calculating these derivatives, we utilize a suitably modified version of the algorithm in Thissen et al. (1995).

**Appendix B**

# Supplementary figures


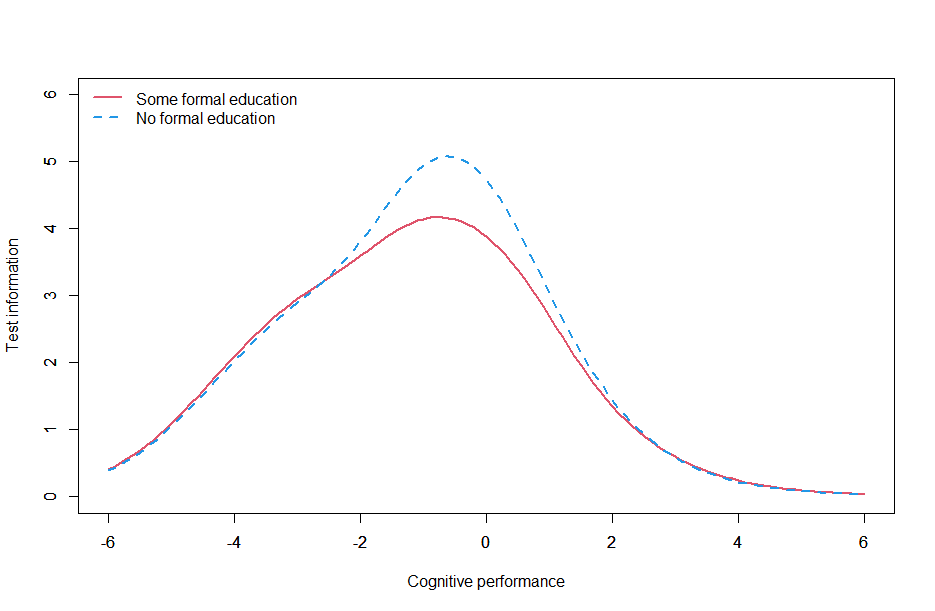


**Figure B1**

Test information function for the two education groups.
